# Secretion of functional human enzymes by *Tetrahymena thermophila*

**DOI:** 10.1186/1472-6750-6-19

**Published:** 2006-03-16

**Authors:** Thomas Weide, Lutz Herrmann, Ulrike Bockau, Nadine Niebur, Ingo Aldag, Wouter Laroy, Roland Contreras, Arno Tiedtke, Marcus WW Hartmann

**Affiliations:** 1 Cilian AG, Johann-Krane-Weg 42, D-48149 Münster, Germany; 2 Institut für allgemeine Zoologie und Genetik, Universität Münster, Schlossplatz 5, D-48149 Münster, Germany; 3 Department of Molecular Biology Research, Unit for Fundamental and Applied Molecular Biology, Ghent and Flanders Interuniversity Institute for Biotechnology, Technologiepark 927, B-9052 Ghent, Belgium

## Abstract

**Background:**

The non-pathogenic ciliate *Tetrahymena thermophila *is one of the best-characterized unicellular eucaryotes used in various research fields. Previous work has shown that this unicellular organism provides many biological features to become a high-quality expression system, like multiplying to high cell densities with short generation times in bioreactors. In addition, the expression of surface antigens from the malaria parasite *Plasmodium falciparum *and the ciliate *Ichthyophthirius multifiliis *suggests that *T. thermophila *might play an important role in vaccine development. However, the expression of functional mammalian or human enzymes remains so far to be seen.

**Results:**

We have been able to express a human enzyme in *T. thermophila *using expression modules that encode a fusion protein consisting of the endogenous phospholipase A_1 _precursor and mature human DNaseI. The recombinant human enzyme is active, indicating that also disulfide bridges are correctly formed. Furthermore, a detailed N-glycan structure of the recombinant enzyme is presented, illustrating a very consistent glycosylation pattern.

**Conclusion:**

The ciliate expression system has the potential to become an excellent expression system. However, additional optimisation steps including host strain improvement as wells as measures to increase the yield of expression are necessary to be able to provide an alternative to the common *E. coli *and yeast-based systems as well as to transformed mammalian cell lines.

## Background

Throughout the last decades the ciliate *Tetrahymena thermophila *has been used as the model system of choice in many areas of molecular, cell and developmental biology [1-4]. For example milestone discoveries like ribozymes that enable RNA-mediated catalysis [5], the basic analysis of dynein motors [6], the finding of telomeres and telomerases [7,8] and the function of histone acetyltransferases in transcription were made in *Tetrahymena*. Very recently, the discovery of small scan RNA elucidates the role of RNAi mediated genome rearrangement [9].

In previous experiments *T. thermophila *has been used to express proteins of two phylogenetic closely related alveolate species. Gaertig *et al*. showed expression of the I-antigen of the ciliate *Ichthyophthirius multifiliis*, a parasite that causes the white spot disease of freshwater fish [10,11]. Later on it could be demonstrated that the GPI-anchored circumsporozoite (CS) protein of the malaria parasite *Plasmodium falciparum*, was expressed and targeted to the surface of *T. thermophila*. Consequently, *T. thermophila *could also play an important role in strategies for vaccine development [12]. All three protozoans *Tetrahymena thermophila, Ichthyophthirius multifiliis *as well as the malaria parasite *Plasmodium falciparum *belong to the alveolates, a distinct phylogenetic group that includes ciliates, apicomplexans and dinoflagellates. They are characterised by both very AT-rich genomes and an unusual codon usage [13-16]. However, so far the expression of functional mammalian or human proteins in *T. thermophila *remains to be shown.

The *T. thermophila *genome has just been completely sequenced by an NIH program and can now be used for thorough proteome analysis (The Institute for Genomic Research [17]). Furthermore, it is known that *T. thermophila *cells grow fast to high cell densities in inexpensive media and simple bioreactor infrastructure [18,19]. Overall, these are the basis for developing an excellent expression system and using the ciliate *T. thermophila *for biotechnology applications [20]. In contrast to the previously done expression experiments here we report for the first time the heterologous expression of a human enzyme that subsequently is secreted into the surrounding medium.

We selected the human DNaseI to demonstrate that *T. thermophila *as an expression host is able to produce functional human enzymes. Human DNase I is a 29.3 kD glycoprotein with two N-glycosylation sites at Asn_18 _and Asn_106 _and two calcium ion binding sites. One of two intrachenar disulfide bridges (Cys_101_/Cys_104 _and Cys_173_/Cys_209_) is crucial for enzyme activity [21]. Aging of the beta-sandwich shaped enzyme is accompanied by deamidation of Asn_74 _thereby reducing enzyme activity and facilitating degradation of the protein [22]. Interestingly, recombinant human DNase I produced in CHO cells (Pulmozyme^®^, Roche) is administered to patients with cystic fibrosis by inhalation in order to reduce sputum viscoelasticity and to improve lung function.

## Results

### Expression and secretion of functional human DNase I by *T. thermophila*

Aim of this study was to show that *T. thermophila *is capable of expressing a human enzyme which is secreted under the control of an endogenous *T. thermophila *derived precursor peptide. The codon bias of ciliates has been analysed in detail [13,15,16]. In summary it is quite different from human genes. 15 of the 63 possible codons in human DNase I are very rare codons in *T. thermophila *according to the definition of Wutschick and Karrer: A rare codon is a codon that is no more than 10% of the total and less than 50% of the fraction expected if all synonyms are used at equal frequency. As an example, codons that encode arginine illustrate this problem. From six possible codons only AGA is preferentially used in highly expressed genes (96%) in *T. thermophila*. The other five codons AGG, CGA, CGC, CGG and CGU are only used in less than 5% of the total arginine codons and especially the CGG codon is very rare. The original human DNaseI sequence (accession number: NM_005223) consists of 14 arginines, but only two of the human original codons are optimal for expression in the *T. thermophila *host system. As both groups Wutschick and Karrer as well as Larsen *et al*. report a correlation between frequently used codons and a higher expression level of genes that contain these codons we used a synthetic "codon-adapted" DNaseI gene to avoid problems in expression.

**Figure 1:**
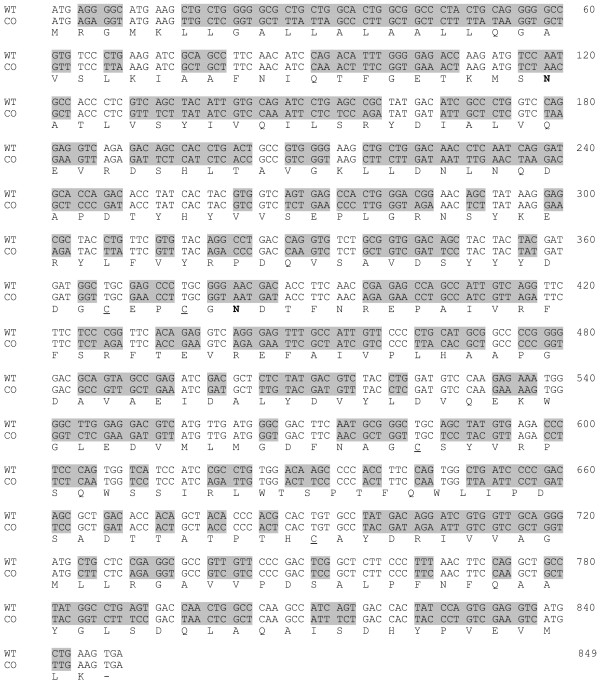
**Codon adaptation of the used synthetic DNase I gene**. As the codon bias of *T. thermophila *is different from that of human genes 67% of 283 coding triplets of the original human codons (WT) were changed into codons that are known to be mostly present in highly expressed *T. thermophila *genes (CO) [13,16]. The adapted codons are marked by gray boxes. The corresponding amino acid sequence is given in single letter code; N-glycosylation sites are highlighted in bold letters, cysteines forming disulfide bridges are underlined.

**Figure 2:**
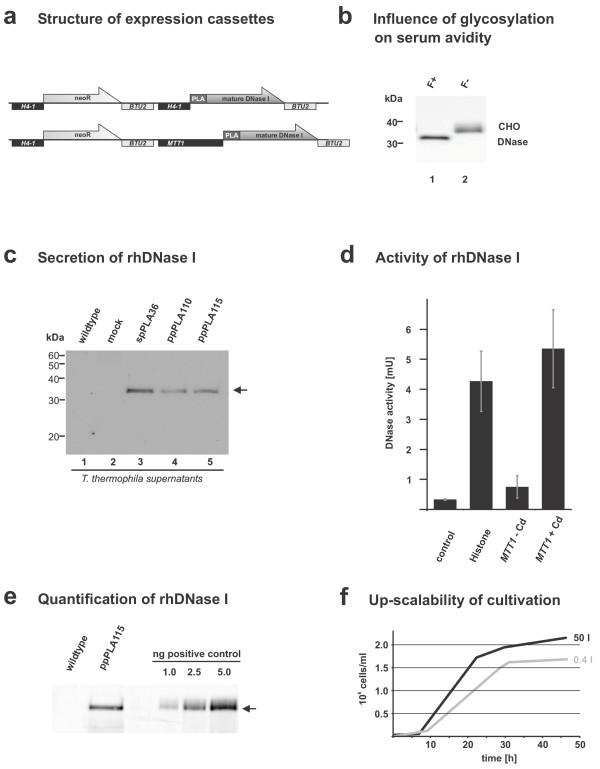
**Expression of functional human DNaseI**. **a**: The structure concept of the expression cassettes. For all expression cassettes we used the cell-cycle dependent *H4-1 *histone or the Cd-inducible MTT1 promotor and the beta tubulin 2 (*BTU2*) terminator sequence. Selection was done by the neo resistance gene (neoR). Secretion of the rhDNase was regulated by parts of the endogenous precursor of PLA_1_. We used the first 36, 110 and 115 amino acids of the PLA-precursor. The mature human DNase I corresponds to the aa 23–281. **b**: We obtained an anti DNaseI serum by using CHO derived DNase I (Pulmozyme) as an antigen. After that the serum was tested by western blot using de-glycosylated DNaseI (PNGaseF treated, F+ lane1) as well as the glycosylated protein. No significant difference in the signal strength could be observed, suggesting that the N-glycan structure of rhDNase has no, or only a minor influence on the avidity of the serum. **c**: Secretion of processed recombinant human DNaseI into the media. *Lane1*: 20 μl supernatant of the *T. thermophila *wildtype strain B1868.7, negative control; *Lane2*: 20 μl supernatant of mock-(EGFP) transformed *T. thermophila*; *Lane 3–5*: 20 μl supernatant of *T. thermophila *cells that were transformed with expression plasmids, carrying the spPLA_36_, ppPLA_110 _and ppPLA_115 _prepro-peptides, respectively. **d**: Determination of DNase activity in 10 μl supernatant of transformed *T. thermophila*. Control: mock-(EGFP) expressing cells; Histone: cell cycle dependent expression of ppPLA_115_; *MTT1*: inducible expression of ppPLA_115_; (*MTT1*-Cd: non-induced; *MTT1 *+ Cd: induced). **e**: Different amounts of rhDNaseI from CHO cells (1.0, 2.5 and 5.0 ng) were used to roughly quantify the yield of rhDNase in 30 μl ppPLA_115 _supernatant of *T. thermophila*. **f**: Demonstration of up-scalability by comparing cell titres during 50 litre fermentation (black) and 400 ml shaker flask cultivation (gray) of rhDNase I secreting *T. thermophila*.

The synthetic optimised gene encodes the human 282 amino acids (aa) of the human precursor of DNase I (accession number: DQ073047). In summary 67% of all codons (188 of 282) were changed into codons that are frequently used in *T. thermophila*. From the 188 optimised codons more than 50% (97 of 188) are changed at the third position. Only one third (93 of 282) of the original sequence seemed to be suitable to express a human enzyme in the ciliate. The original human DNase I cDNA and the codon adapted sequence are compared in figure 1. In our experiments we want to express and secrete the human DNaseI by a ciliate system. Therefore we constructed expression cassettes that encode parts of the precursor sequence of the PLA_1 _(phospholipase A_1_) gene (accession number: AJ508393) and the mature human DNaseI (aa 23 to 281) of the synthetic gene [23]. The PLA_1 _prepro-peptide has significant similarity to members of the cathepsin L family and mediates secretion into the medium [24]. We have previously characterized the cleavage site between the pro-peptide and the mature enzyme to be between aa 110 and 111 by sequencing of the N-terminus of the mature PLA_1 _enzyme (unpublished data). The first fusion protein encodes the PLA_1 _signal peptide as determined by SignalP [25] (aa 1–23 of PLA_1_), an additional 13 amino acid spacer (aa 24–36 of PLA_1_) and the mature DNaseI (aa 23 to 281 encoded by the synthetic gene). The second hybrid protein consists of the whole prepro-peptide of PLA_1 _(aa 1–110) and the mature DNaseI. The third cassette is similar to the second except for the first five amino acids of the secreted mature PLA_1 _(single letter code GEATE; aa 111–115). This cassette was made to ensure an optimal cleavage of the proPLA_1_-DNaseI fusion protein by endogenous pro-peptidases. The expression cassettes were cloned into the pH4T2 vector that consists of two rDNA origins and the neomycin resistance gene (*neoR*) [26]. Both the neoR gene and the PLA_1_-DNasehybrid genes were controlled by the cell-cycle dependent histone promoter *H4-1 *and the beta tubulin 2 *BTU2 *terminator (Figure 2a). We transformed conjugating *T. thermophila *with the expression plasmids by electroporation, and monitored the successful transformation by selection against paromomycin. Supernatants of the different cultures were analysed by SDS-PAGE and Western blot for secretion of the recombinant human DNaseI (rhDNaseI). All samples analysed expressed and secreted rhDNaseI, regardless of the length of the PLA_1_-prepro-peptide sequence fused to the DNaseI (Figure 2c). Furthermore, all expression modules resulted in rhDNaseI proteins of the same size (~35 kD), indicating the existence of a processing mechanism that is independent of a conserved pro-peptidase recognition site, similar to the known processing of granule lattice proteins of dense core vesicles in *T. thermophila *[27]. A control strain that expresses and secretes EGFP and the non-transformed *T. thermophila *wild type strain B1868.7 was used. Supernatants of cells expressing the ppPLA_115_-DNase fusion protein were assayed for DNase activity. Compared to supernatants of mock-transformed cells a significantly elevated enzyme activity was measured. The heterologous expression of human DNaseI by *T. thermophila *was also analysed by application of the inducible metallothionein-1 promoter (*MTT1*) system [28] (Figure 2d). In these experiments the expression of the ppPLA_115_-DNase fusion protein was regulated by the addition of Cadmium to the medium. The induced cells exhibited a significantly elevated DNase activity in the supernatant. In non-induced cultures the DNase activity remained – just like expected – close to the basal level. This clearly argues that the observed increased DNaseI activity in the supernatant is due to the secretion of functional rhDNase, confirming the experiments shown in Figure 2c. The basal activity measured in the negative control corresponds to at least two endogenous DNases (data not shown). As rhDNase combines in one enzyme all assets necessary for development of a novel expression system with secretion, disulfide bridge formation and glycosylation site recognition the main focus of our initial experiments was to illustrate that not only antigens from related species (*I. multifiliis *and *P. falciparum*) but also functional human enzymes of biotechnological interest can be expressed and secreted by the ciliate system. Nevertheless, to give a first and roughly estimation of the yield of secreted rhDNase we used semi-quantitative Western blotting. Therefore we generated a polyclonal antiserum against the rhDNaseI from CHO cells and tested first if the N-glycosylation has an impact on avidity. Figure 2b clearly illustrates that both glycosylated DNaseI as well as the de-glycosylated enzyme display the same signal strength in Western blot analysis, suggesting that almost all of the antigenic epitopes recognised are on protein level and that the N-glycan structure plays an unimportant role in avidity. This allows us to give a first and rough estimation about the amount of the secreted DNaseI, by semi-quantitative Western blotting. We found ca. 100 μg rhDNase per litre supernatant fluid (see Figure 2e; 1 ng/10 μl supernatant). This crudely estimated amount combined with the determined DNase activity of 400 U per litre supernatant (4 mU/10 μl, see Figure 2d) means that a "specific" activity can be determined as ca. 4 Units per μg rhDNase. For most biotechnical applications it is crucial to obtain a first impression whether or not the cultivation of transformants can be scaled up. Therefore we tested if the rhDNA secreting clone is growing in the same way as previously described for wildtype strains [20,29]. RhDNase I expressing cells were grown on a small scale (400 ml shaker flask), as well as in 2 and 50 litre batch fermentations. Figure 2f illustrates that growth (up to more than 2 × 10^6 ^cells/ml) of transformants is independent of the culture volume. Furthermore up-scaling does not alter results shown in Figure 2b–d.

### Glycosylation pattern on recombinant proteins derived from *T. thermophila*

**Figure 3:**
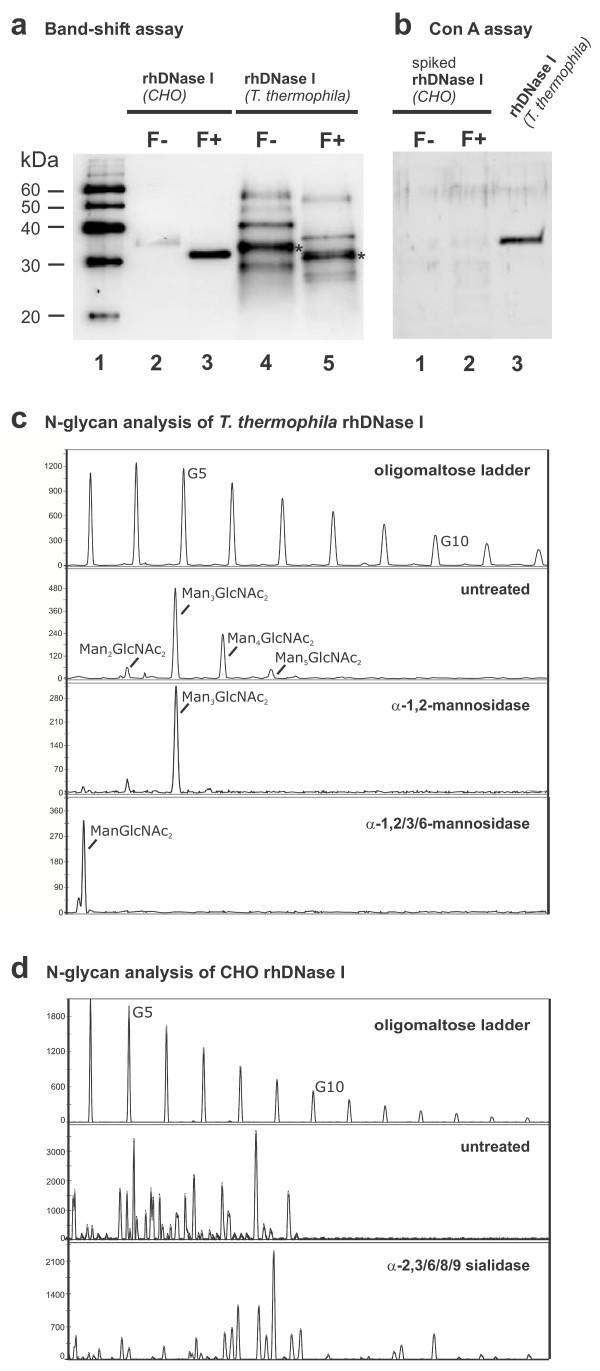
**N-glycan structure on recombinant secreted human DNase I**. **a**: Band-shift assay: rhDNase I from CHO cells and secreted rhDNase I from *T. thermophila *were de-glycosylated. The treatment with PNGase F (F+) results in a significant band-shift, when compared to the controls (F-), illustrating that the secreted rhDNase I of *T. thermophila *becomes glycosylated. As *T. thermophila *rhDNase I had been concentrated by immunoprecipitation, additional bands of antibody-chains appear in Western blotting. The DNase signals are highlighted by asterisks. **b**: Concanavalin A pulldown assay: Supernatants of transformed *T. thermophila *strain and a wildtype strain (B1868.7) were analysed. The supernatant of the wildtype was spiked with equal amounts (2 μg) of glycosylated (F-) or de-glycosylated (F+) rhDNase I from CHO cells, respectively. Only mannose-terminated glycosylated rhDNase is recovered by Con A (lane 3), glycosylated (F-) and de-glycosylated rhDNase I (F+) from CHO cells did not bind to ConA beads (lanes 1 and 2). **c**: DSA-FACE N-glycan analysis of *T. thermophila *rhDNase I: This data illustrates the highly consistent N-glycan structure on rhDNaseI produced in *T. thermophila*. All N-glycans are mannose terminated, as shown by digestion with jack bean mannosidase in the lower panel. The most prominent structure is the Man_3_GlcNAc_2 _core pattern with additional alpha 1,2 bound mannoses (two middle panels). **d**: DSA-FACE N-glycan analysis of CHO rhDNase I: On the CHO derived rhDNase I more than 20 distinct peaks, each corresponding to a different N-glycan, are observed in the untreated sample. This typical complex profile of mammalian cells is in sharp contrast to the consistent *T. thermophila *glycosylation. Even removal of terminal N-acetylneuraminic acid by treatment with sialidase still leads to a large number of various glycans (lower panel).

Transport through the ER and Golgi compartments usually is accompanied by the addition of N-glycans. We addressed this point for the recombinant DNaseI and performed band-shift assays by applying N-glycosidase F (PNgase F) to immunoprecipitated DNaseI from supernatant of transformed *T. thermophila *cells (Figure 3a). An aliquot of this IP sample (F+) revealed a significant band-shift on a 15% SDS-gel when compared to the control without N-glycosidaseF treatment (F-). Glycosylated and de-glycosylated DNaseI from CHO cells was used as control. The significant reduction of the molecular weight of secreted rhDNaseI from *T. thermophila *demonstrates that the DNase becomes glycosylated while shuttling through the ER and Golgi. Furthermore comparison of lane 3 and 5 also shows that the non-glycosylated rhDNase from *T. thermophila *and CHO-cells are running at heights of comparable molecular weight. Next we applied a Concanavalin A (Con A) pulldown assay to test whether the glycans of secreted rhDNaseI are terminated by N-terminal mannoses, as described for endogenous secreted enzymes of *Tetrahymena *[30,31]. We used the same cell-free supernatants as in the IP approach (compare Figure 3a lane 4 and 5). The B1868.7 wildtype supernatant spiked with glycosylated and de-glycosylated rhDNaseI from CHO cells was used as control. We added ConA beads and performed extensive washes, to avoid unspecific binding. Only the rhDNaseI from *T. thermophila *was recovered by the ConA beads, indicating that it predominantly contains α-Dmannose terminated glycosylation chains. The DNaseI from CHO cells which contains a different, complex N-glycan feature with terminal galactose, N-acetylglucoseamine or sialic acid residues and the de-glycosylated rhDNaseI did not bind to the ConA (Figure 3b). The specificity of the ConA pulldown assays was nicely confirmed by the DSA-FACE technology for N-glycan profiling [32]: These results illustrate that almost all recombinant protein displays a mannose terminated oligo-glycosylation pattern, comparable to endogenous secreted proteins [30]. The most prominent N-glycan is the Man_3_GlcNAc_2 _structure (Figure. 3c). All structures can be reduced to ManGlcNAc_2 _using an unspecific mannosidase, indicating the presence of mannose only at the core. Overall the *T. thermophila *pattern is very consistent (see Figure 3c, second panel). This consistency is even more obvious when compared to the typical complex glycosylation of rhDNase I from CHO cells shown in Figure 3d (see second and third panel).

## Discussion

Here we report for the first time the expression and secretion of functional human DNaseI, illustrating that not only surface proteins from related species but also mammalian proteins are potential candidates. To avoid problems in expression due to the fact that the codon bias in *T. thermophila *is quite different to that of mammalian cells we used a codon adapted gene in which critical triplets were changed (see figure 1). All hybrid constructs used lead to expression of the rhDNaseI in *T. thermophila*. These results clearly show that the here presented ciliate system has the high potential to become an attractive alternative for expression and secretion of complex functional human proteins. Furthermore, unlike in yeast expression systems, where hyper-glycosylation causes problems on recombinant proteins, the rhDNase secreted by *T. thermophila *carries a consistent, oligo-glycosylated N-glycan structure [33,34]. Additionally, the predominant Man_3_GlcNAc_2 _sequence on *T. thermophila *derived proteins could be an interesting starting point for *in vitro *glycosylation to produce homogenous glycoproteins. A well known problem of bacterial expression systems is the proper formation of disulfide bridges. As the expression and secretion of rhDNase I in the free-living protozoan *T. thermophila *yields a highly active enzyme, at least the required internal disulfide bridge (Cys_173_-Cys_209_) must have formed in the right manner [21].

## Conclusion

The roughly estimated yield of 100 μg/L rhDNase I in *T. thermophila *is a promising starting point for further improvements of this very young expression system. Established and commonly known systems surely currently have higher expression rates (e.g. 5 mg/l rhDNaseI in COS-cells [35]). Yet there are other criteria to be met for a new expression host to be adopted by the biotech industry like up scalability, simple and inexpensive media, growing to high cell densities, protease-deficiency and ease of cell line engineering of the production strains that all contribute to low cost of manufacturing. In *T. thermophila *these requirements can be fulfilled because a simple bioreactor infrastructure is sufficient for production. Moreover, high cell density fermentation with cell retention is available in our laboratories [18,29]. The use of such techniques allows us to obtain cell densities in inexpensive media of more than 2.2 × 10^7 ^cells/ml, equivalent to 48 g dry weight, thereby reducing the cost of manufacturing. Genetic tools are at hand to increase promoter activity and to improve strains easily by targeted knock outs of proteases, random mutagenesis and secretion optimisation. These measures will lead to an excellent expression-secretion system bearing a competitive alternative to the common and technically mature systems like *E. coli*, yeast and transformed mammalian cell lines.

## Methods

### Strains, cultivation and fermentation

*Tetrahymena thermophila *strains B1868.4, B1868.7 and B 2068.1 were kindly provided by Peter J. Bruns and cultivated in skimmed milk medium (2% skimmed milk, 0.5% yeast extract, 0.1% ferrous sulphate chelate solution and 1% glucose) on a Braun Certomat BS-1 at 80 rpm and 30°C. For fermentations a Braun UD50 (50 litre) and Bioengineering KLF2000 (2 litre) equipped with standard Rushton impellers were used. Stirrer speed was limited to 300 and 400 rpm respectively; pO_2 _was set to 25%.

### Generation and transformation of expression plasmids

Transformation of cells was performed with plasmids derived from the pH4T2 vector [26]. For the Cd-inducible expression the histone promoter in the ppPLA_115_-construct was substituted by the *T. thermophila *MTT1-promoter. Metallothioneins, that are upregulated upon stress, are metal binding proteins playing a role in detoxification of the cell. In *Tetrahymena *the MTT1-promoter can be induced best by the addition of Cadmium [28]. Cloning of the expression modules and mutagenesis were performed with standard techniques. To ensure a translation of the fusion proteins a synthetic, codon optimised human DNaseI gene was used [13,15]. The synthesis was performed by a solid phase process described in German Patent DE 19812103.2 by the company ATG biosynthetics, Merzhausen Germany. In brief oligonucleotides were specifically annealed 3' at the immobilized single stranded DNA and extended by a polymerase. A double strand-specific 5' nuclease digestion enables subsequent annealing of a new primer 3' at the growing synthetic gene and the cycle starts over. The sequence has been submitted to genbank (Accession number: DQ073047).

### Generation of polyclonal anti human DNase I antibodies from rabbit

Recombinant human DNaseI from CHO cells (Pulmozyme^®^, Roche) was used to generate a specific antiserum from rabbit against human DNaseI. Affinity purification using a protein A/protein G mixture was performed in order to minimize background signals.

### SDS-PAGE and Western

The aliquots of SPP supernatants were resuspended in sample buffer and separated on 15% SDS-PAGE. rhDNaseI from CHO cells (Pulmozyme^®^, Roche) served as reference. The gels were blotted onto nitrocellulose membranes and blocked in PBS containing 0.05% Tween20 and 5% skimmed milk (PBS-TM). The anti-rhDNaseI was used in a 1:500 dilution in PBS-TM. After washing with PBS/T and application of HRP-conjugated anti rabbit serum the blots were developed using chemiluminescence.

### Immunoprecipitation of DNase I from *T. thermophila *supernatants

Anti DNase serum was coupled to cyanogen bromide activated Sepharose 4B according to the manufacturer's instructions. *T. thermophila *supernatants were applied to the column. After washing with PBS, bound protein was eluted with 0.1 M HCl-glycin pH2.8 and neutralized with 2 M Tris.

### DNase I activity assay

The methyl green based DNase activity assay was performed as already published [36]. Samples were incubated at 37°C for 24 h on a microtiter plate. Absorbance was measured at 620 nm.

Calibration of the assay was achieved by different amounts of defined DNase I Units of Pulmozyme^® ^from Roche (CHO derived) in each experiment and linear regression. These results combined with semi-quantitative western blotting were used to calculate the specific activity of expressed DNase I.

### Purification of rhDNase I and N-glycan analysis of rhDNase I from recombinant *T. thermophila *supernatants

Purification of the rhDNase I was performed with slight modifications as described previously [35]. *Band-shift assay*: We performed an immunoprecipitation as described above. An aliquot of the IP sample was de-glycosylated by applying N-glycosidaseF (Roche, Germany) according to the manufacturer's instructions. The de-glycosylation of rhDNaseI was used to control the glycosylation assay. *Concanavalin pull down assay*: We used ConA coupled to Sepharose 4B beads. The beads were washed and resuspended in PBS in order to prepare a 50% slurry. 200 μl of the 50% slurry was added to cell-free supernatant. The beads were incubated for 3 h under rotation at room temperature to allow the Con A beads to bind to the glycosylated proteins. After that, Con A beads were collected by centrifugation (2 min, 1000 × g at 4°C) and subsequently washed in PBS with 1% TritonX-100. All samples were analysed by SDS-PAGE and Western blot. *DSA-FACE analysis of N-glycans*: Purified recombinant DNaseI from transformed *T. thermophila *and rhDNase I from CHO cells were analysed by the DSA-FACE N-glycan analysis method, as previously described [32]. Enzymatic digestions (*T. reesei *alpha 1,2-mannosidase, jack bean mannosidase and *Arthrobacter ureafaciens *alpha 2,3/6/8/9-sialidase) were done in 20 mM NaAc, pH 5.5 for 16 h.

## Authors' contributions

TW participated in the cloning, western blots and the Con A pulldown assays, carried out conception and manuscript drafting. LH carried out the immunoprecipitation, purification and DNase I assays and participated in conception and manuscript drafting. UB participated in the cloning of expression constructs and performed the transformation of the Ciliates. NN cloned expression constructs. IA was responsible for fermentations and media compositions. WL did the DSA-FACE analysis. RC made the conception for the N-glycan profiling. AT helped to draft the manuscript. MWWH conceived of the study and participated in its design and coordination. All authors read and approved the final manuscript.
